# Temporal Co-Variation between Eye Lens Accommodation and Trapezius Muscle Activity during a Dynamic Near-Far Visual Task

**DOI:** 10.1371/journal.pone.0126578

**Published:** 2015-05-11

**Authors:** Camilla Zetterberg, Hans O. Richter, Mikael Forsman

**Affiliations:** 1 Centre for Musculoskeletal Research, Department of Occupational and Public Health Sciences, Faculty of Health and Occupational Studies, University of Gävle, Gävle, Sweden; 2 Department of Medical Sciences, Section of Occupational and Environmental Medicine, Uppsala University, Uppsala, Sweden; 3 Institute of Environmental Medicine, Karolinska Institutet, Stockholm, Sweden; Washington University, UNITED STATES

## Abstract

Near work is associated with increased activity in the neck and shoulder muscles, but the underlying mechanism is still unknown. This study was designed to determine whether a dynamic change in focus, alternating between a nearby and a more distant visual target, produces a direct parallel change in trapezius muscle activity. Fourteen healthy controls and 12 patients with a history of visual and neck/shoulder symptoms performed a Near-Far visual task under three different viewing conditions; one neutral condition with no trial lenses, one condition with negative trial lenses to create increased accommodation, and one condition with positive trial lenses to create decreased accommodation. Eye lens accommodation and trapezius muscle activity were continuously recorded. The trapezius muscle activity was significantly higher during Near than during Far focusing periods for both groups within the neutral viewing condition, and there was a significant co-variation in time between accommodation and trapezius muscle activity within the neutral and positive viewing conditions for the control group. In conclusion, these results reveal a connection between Near focusing and increased muscle activity during dynamic changes in focus between a nearby and a far target. A direct link, from the accommodation/vergence system to the trapezius muscles cannot be ruled out, but the connection may also be explained by an increased need for eye-neck (head) stabilization when focusing on a nearby target as compared to a more distant target.

## Introduction

Near work, e.g. working on a computer, is associated with musculoskeletal disorder and pain in the area of the neck and shoulders [1–5]. Despite a number of ergonomic intervention studies focusing on reducing these problems (evaluating for example work station design, input devices, ergonomic training, optometric corrections, and lighting), these problems still remains [6–9]. Previous experimental investigations indicate that the visual load required when working on a computer in some way enhances general muscle activity in this area [10–12].

Two of the previous experimental investigations required the subjects to focus on a nearby target continuously, without interruption, for five and seven minutes respectively. The first investigation revealed a positive general correlation between eye lens accommodation and trapezius muscle activity [11]. The second study also revealed a positive correlation between accommodation and trapezius muscle activity, but the correlation was significant only when incongruence between accommodation and convergence was present [12]. These studies were designed to investigate the effect of sustained focusing on a nearby target on trapezius muscle activity, and it was not possible to evaluate if dynamic changes in eye lens accommodation between a nearby and a far target produces a direct parallel change in trapezius muscle activity.

Since eye lens accommodation is driven by activity in the ciliary muscles, a direct parallel change in trapezius muscle activity due to changes in eye lens accommodation could hypothetically involve the ciliary muscles. When clear vision is acquired an efferent signal is sent to the ciliary muscles to alter the refractive power of the eye lens; a copy of this signal could be sent to the trapezius muscles and/or other muscles in the neck/shoulder area in order to stabilize gaze (cf. [11]).

Patients with visual symptoms often report discomfort and pain in the neck/shoulder area [13]. In school children, accommodation- and convergence insufficiency is associated with increased visual and musculoskeletal symptoms [14]. If persons with a history of visual- and neck/shoulder symptoms respond differently to visual demands than healthy controls has not yet been fully explored [15,16].

The purpose of this study was to investigate whether a dynamic change in focus produces a direct parallel alteration in trapezius muscle activity. An experimental study was designed with a Near-Far visual task where subjects alternately focused on a nearby and on a more distant target requiring more and less extensive eye lens accommodation, respectively. The Near-Far task was repeated three times under different viewing conditions; one condition with no trial lenses, one condition with negative trial lenses to create increased accommodation, and one condition with positive trial lenses to create decreased accommodation. The hypotheses were:

1. Trapezius muscle activity is higher when focusing on a nearby than on a more distant target, therefore
  - the subjects’ trapezius muscle activity amplitude should be higher during a period of near focusing than during a neighbouring far focusing period,
  - there should be a positive co-variation in time between accommodation and trapezius muscle activity during the Near-Far task.
2. For viewing conditions requiring more eye lens accommodation, i.e. more activity of the ciliary muscles, the effect on trapezius muscle activity and the co-variation in time described in hypothesis (i) should be more pronounced.

The hypotheses were tested separately on healthy controls and on patients with a history of visual and neck/shoulder symptoms.

## Methods

Data for this study were collected as a part of a larger study examining visual stresses on musculoskeletal loads [11]. This report includes three of the four viewing conditions studied in conjunction with a Near-Far visual task. For further details on the overall methods, see Richter et al. (2010) [11].

### Subjects

Seventeen women and nine men with a mean age of 29 years (range 19–42, SD 8) participated in this study. Fourteen of these subjects were healthy and formed the control group. Twelve had a history of eye disorders (asthenopia) and non-specific neck disorders and formed the patient group. The age distribution within groups is presented in Table 1.

**Table 1:** Subjects.

	Controls (n = 14)			Patients (n = 12)		
	6 men, 8 women			3 men, 9 women		
	Mean	Range	SD	Mean	Range	SD
Age	26	19–42	8	32	21–42	7
NPA (in D)	10.3	6.8–11.7	1.8	8.8	6.3–11.7	1.8
DNT (in cm)	23.1	15–55	13.3	33.6	15–80	19.8
DNT (in D)	5.3	1.8–6.7	1.8	3.8	1.3–6.7	1.8
NDI	-	-	-	9.3	3–20	4.5

Age distribution, near point of accommodation (NAD) and distance to near target (DNT) for the two groups, and results of the Neck Disability Index scale (NDI score 0–50) for the patient group. D = diopters.

All subjects in the control group underwent a regular optometric examination and were deemed to have normal unaided or aided vision, normal near point of accommodation and convergence, and no history of eye disease.

The patient group had been referred to an orthoptist for investigation and treatment. The orthoptic investigation included tests of stereoscopic vision, strabismus, near point of accommodation and convergence, and a complete refraction in cycloplegia. Eye disorders in the patient group ranged from uncorrected refractive errors (hypermetropia, myopia, astigmatism and anisometropioa) and insufficient accommodation, to neuromuscular anomalies such as heterophoria, heterotropia, and insufficient convergence. All significant refractive errors found by the orthoptist were corrected for using spherical and cylindrical glasses, all orthoptic treatment were completed and all patients were free from visual symptoms in the two to three months prior to study participation. At the time of the study, all patients rated some neck problems using the Neck Disability Index (NDI) (Table 1). The NDI (score 0–50) is an instrument for assessing self-rated disability in patients with neck pain, and has been used in both clinical and research settings [17,18]. Only subjects with a history of asthenopic symptoms, and concurrent self-reported neck problems were defined as patients.

### Ethics statement

All subjects were given verbal and written descriptions of the experimental protocol and signed informed consent form prior to participating in the study. The study was approved by the Medical Ethical Review Board of Uppsala University (2006:027), and was conducted according to the Declaration of Helsinki.

### Experimental set-up

**Fig 1:**
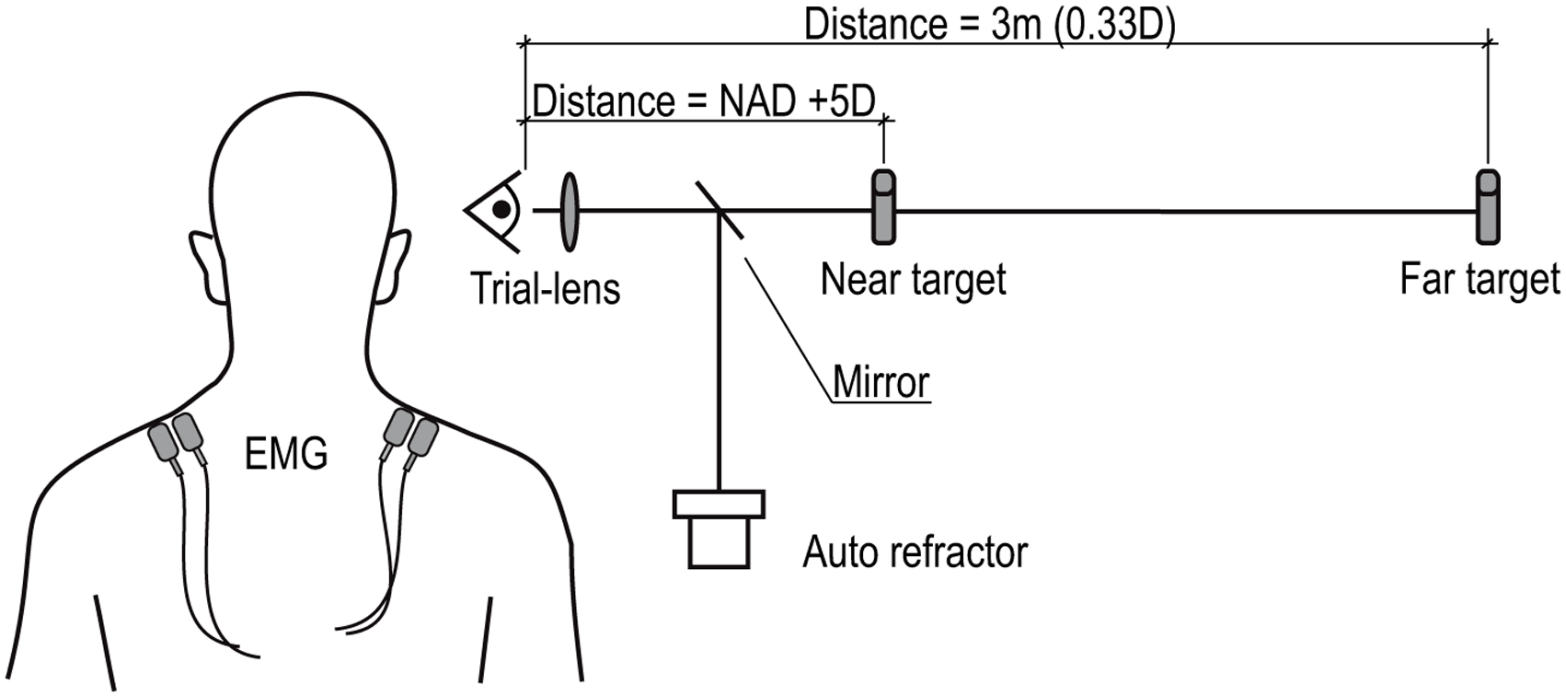
The laboratory set-up. This is a schematic illustration of the experimental set-up in the laboratory. The distance to the Far target was 3 m (0.33 D) for all subjects. The distance to the near target was individually adjusted to be 5 D away from each individual’s near point of accommodation (NAD + 5 D). D = diopters.

The study protocol began by assessing subjects, binocularly, for refractive errors using an auto refractor (Power Refractor R03, Plusoptix, Nürnberg, Germany) [19]. Next, subject were set-up with surface electromyography (EMG) electrodes bilaterally on the descending portion of the upper trapezius muscles using disposable Ag-AgCl electrodes (Neuroline 725, Ambu A/S, Ballerup, Denmark) and 0.5% saline-based electrode paste (GEL101, BIOPAC Systems, Inc., Santa Barbara, CA, USA). Pairs of electrodes were centred 20 mm lateral to the midpoint of the line between the spinosus process of vertebra C7 and the acromion process (Fig 1); a grounding electrode was positioned atop the spinosus process of vertebra C7. Before applying the electrodes, the skin was rubbed with abrasive paper and cleaned with alcohol to minimize impedance. Subjects performed three 15 s submaximal normalisation contractions with their arms in 90° abduction in shoulder joint, straight elbows and relaxed wrist joints [20]; trials were separated by 30 s of rest. Electrocardiography (ECG) electrodes were applied laterally to the left and right of the sixth rib (EL503, BIOPAC Systems, Inc., Santa Barbara, CA, USA). Thereafter, subjects were seated in a slightly backwards inclined office chair that had head and back support for the duration of the study. To ensure a sufficiently high number of data points from the auto refractor, subjects were instructed not to move during these tasks.

### Experimental task

Subjects performed a visual Near-Far task under three viewing conditions. The Near-Far task involved focusing back and forth between two targets, a Near and a Far target in a shape of an X (Fig 1). The two targets were brightly illuminated from behind by a polychromatic Light Emitting Diode (LED) (Everlight Electronics Co., Ltd., England). The heights of the Near and the Far targets were consistent for all subjects, and were 0.5 cm and 6.8 cm, respectively. The Near-Far task lasted approximately 2.5 minutes, included 15 Near and 15 Far focusing periods, and was repeated twice for each viewing condition. The time for changing between the Near and Far targets varied randomly from 3–7 s (mean 5 s). The distance to the Near target was individually adjusted to be 5 diopters (D) away from each individual’s near point of accommodation. The Far target was placed at a distance of 3 m (0.33 D) from the subject, regardless of an individual’s near point of accommodation (Fig 1).

An individual’s near point of accommodation (NAD) was determined as follows: the subject’s vision was corrected according to the auto refractor analysis and the Near target was presented at approximately 1 m. The subject was instructed to “…*look at the target*, *keep it clear*, *and indicate when you perceive blur and/or diplopia…*”. The Near target was then moved towards the subject until he/she reported blur and/or diplopia. This was repeated five times and the average stopping point was used as a measure of the individual’s near point of accommodation (expressed in D). The estimated near points of accommodation were, in general, consistent with the expected age appropriate near point. Mean near point of accommodation and mean distance to the Near target is presented in Table 1.

### Viewing conditions

Three viewing conditions were used to create different eye lens accommodation conditions as follows: *Neutral*—no trial lenses, *Negative*—wearing—3.5 D trial lenses to create increased accommodation, *Positive*—wearing +3.5 D trial lenses to create decreased accommodation. The individual spherical refractive error detected with the auto refractor (in steps of 0.25 D) was added to the power of the trial lenses. If the participant did not have any refractive error, a—0.25 D lens was added in order to blind the participant to the different viewing conditions. The trial lenses were then mounted in frames (Oculus Inc., Dutenhofen, Germany) and used during the Near-Far task repeats.

### Data recording and processing

During the Near-Far tasks eye lens accommodation was continuously recorded by the auto refractor at 25 Hz [21,22]. The auto refractor recorded accommodation at 1 m without compensating for working distance, i.e. for an emmetropic eye focused at 1 m the auto refractor registered 0 D of sphere. To obtain a measure of the accommodation that allows comparison between Near and Far viewing and between the different viewing conditions, the recorded accommodation was converted in two steps.

First the default distance (1 m) was shifted to optical infinity:

Accommodation ∞=1 − (auto refractor output) (1)

Thereafter the dioptric power of the trial lenses for the negative and positive viewing conditions was corrected for:

$${\text{Accommodation response = accommodation}}^{\infty }-\left(\text{dioptric power of trial lenses}\right)$$ (2)

This accommodation response (expressed in diopters) provides a measure of the refractive power of the individual’s eye lens. To evaluate the second hypothesis (ii); for viewing conditions requiring more eye lens accommodation, the effect on trapezius muscle activity and the co-variation in time described in hypotheses (i) should be more pronounced; accommodation response was used as an indirect measure of ciliary muscle activity (see [11] for more details on how the auto refractor data were converted).

A moving average was calculated across each Near and each Far focusing periods (15 Near and 15 Far periods per each 2.5-min recording). The most stable 1.5 s window value for each Near and each Far period was saved if the window had a minimum of 15 out of 37 valid samples from the auto refractor. Then, a mean for Near and a mean for Far was calculated, if there were a minimum of 8 out of 15 period values. This process was repeated for each eye (right and left), and for each Near-Far task (2 x 2.5 minutes recording of accommodation), resulting in four estimates of accommodation response for each subject and each viewing condition. Since no differences were found in accommodation response between the first and second 2.5-min recording trial, or between the right and left eye (cf. [11]), a single mean accommodation response value was calculated for each subject and each viewing condition for whom two or more valid estimates of accommodation existed.

For periods containing only one valid signal (from either the right or the left eye), that signal was used for the co-variation analyses in time (i.e. the cross-correlation analyses, see below); in periods with no valid signal, no data were used at all. Signals were band-passed filtered at 0.05–3 Hz. The cut-off frequencies were chosen to filter out variations (noise) that were unrelated to the Near-Far task accommodation.

**Fig 2:**
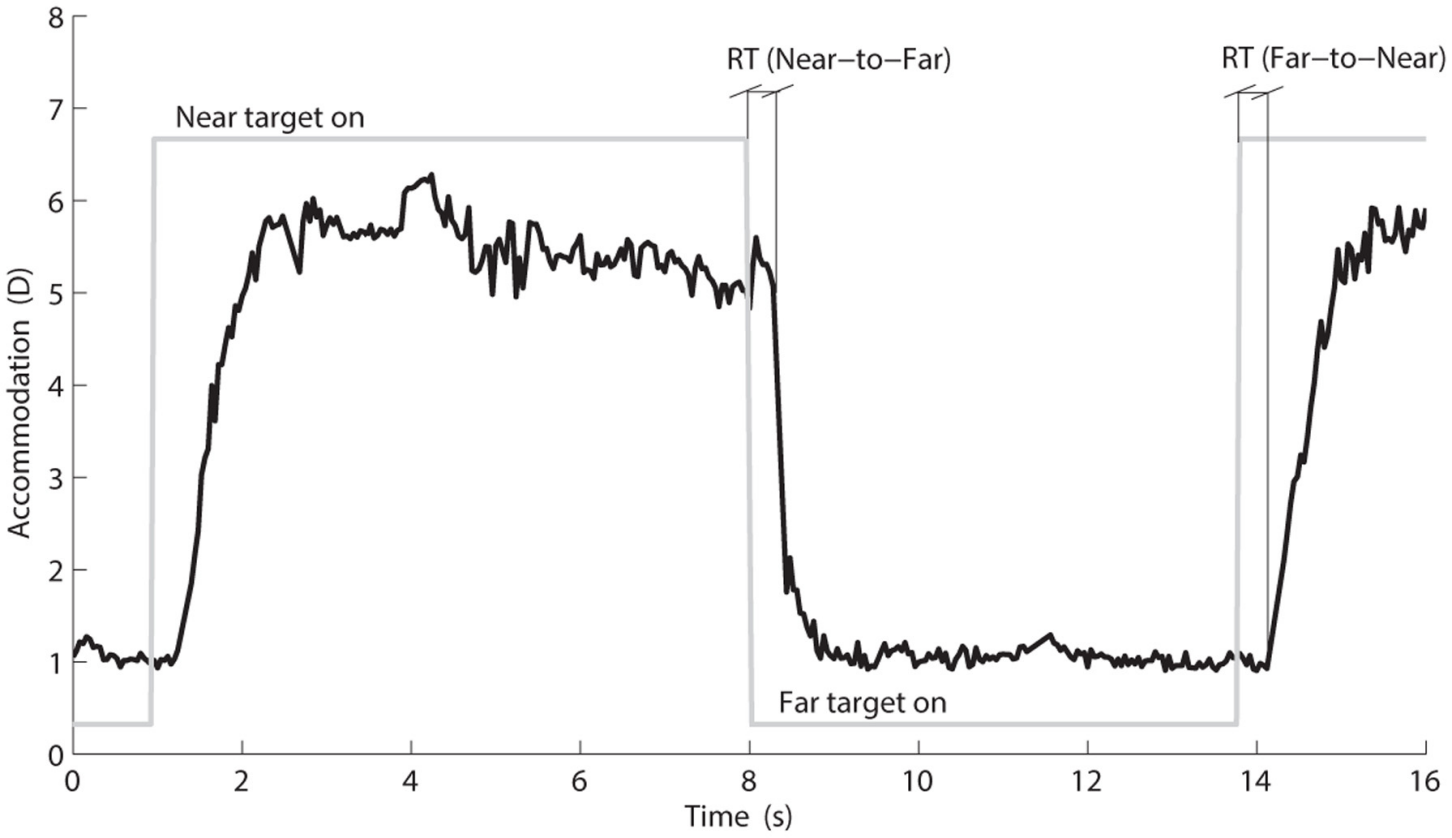
Shift in accommodation from the Near to the Far target. This is a representative shift in accommodation, with the neutral trial lens, from the Near to the Far target and back. The grey horizontal lines show which of the targets that is turned on (Near or Far) and the grey vertical lines depict the shifts between these. RT = Reaction time.

To validate the measurement from the auto refractor and to check that the subjects followed the instructions and changed focus between the two targets, the reaction time (RT) was calculated. RT was defined as the time between the shift in target (when the LED in the Near target turns off, and the LED in the Far target turns on or vice-versa) and the beginning of the shift in eye accommodation (i.e. when the accommodation decreased/increased in comparison with the accommodation level prior to the target shift) (see Fig 2 in the result section). To calculate individual mean RT for the Near-to-Far and the Far-to-Near transitions, the same procedure described above for the mean accommodation responses was used. RTs above 1.5 s were deemed to reflect inattention to the Near-Far task and were therefore excluded.

During the Near-Far tasks EMG and ECG was continuously recorded. EMG and ECG signals were amplified, band-pass filtered (EMG: 10–500 Hz, ECG 0.05–35 Hz), and sampled at 2000 Hz (EMG100C, BIOPAC Systems, Inc., Santa Barbara, CA, USA) during all Near-Far tasks. Noise in the EMG signal caused by heart beats was reduced using the ECG signal as described by Widrow et al. (1975) [23] and modified by Zetterberg et al. (2013) [12]. The EMG recordings were converted into root-mean-square values (RMS) on 0.1 s windows. The noise level was estimated as the lowest 0.4 s moving RMS value of the recording during rest. The noise level was then subtracted from the signal in a power sense, i.e. the squared noise was subtracted from the squared signal and the resulting signal was the square root of this difference. The signal was then normalised to the mean RMS value of the central 10 s of the three 15 s submaximal reference contractions; and expressed as %RVE (reference voluntary electrical activity) [20]. The mean of the RMS EMG across right and left trapezius muscles was computed.

To quantify muscular activity level during each viewing condition, 50^th^ percentile RMS EMG values were computed for Near and Far focusing periods, including values 1 s after target shift until 1 s prior to the next target shift. EMG from Near and Far target focusing was expressed as a percentage of the EMG level during Far target focusing (%Far) for each viewing condition; by this scale Far target focusing periods were 100%Far for all viewing conditions.

Raw EMG signals from each 2.5 min trial during the Near-Far task were band-pass filtered 20–500 Hz, RMS-transformed (0.2 s window), interpolated to match the sampling frequency of the auto refractor signal (25 Hz), and band-pass filtered at 0.05–3 Hz for use in cross-correlation analyses with the auto refractor signals.

### Data analyses

Signal processing was performed using custom software written in MATLAB (MathWorks Inc., Natick, MA, USA). Statistical tests were carried out with SPSS 20.0 for Windows (SPSS Inc., Chicago, IL, USA). The level of significance was set at α = 0.05.

Group (control and patient) mean values for the Near and Far focusing periods in the Near-Far task within each viewing condition were calculated for accommodation response and for trapezius muscle activity (EMG expressed in both %RVE and in %Far). For reaction time, group mean values within viewing condition and group were calculated for Near-to-Far and for Far-to-Near values (see Fig 2 in the result section). Group differences were analysed for each viewing condition using independent sample t-test for: accommodation response (for both Near and Far focusing periods), trapezius muscle activity (in %Far), and reaction times (for both Near-to-Far and Far-to-Near values).

To evaluate whether trapezius muscle activity was higher when focusing on a nearby target than on a more distant target (hypothesis (i)), two analyses were performed:

- To test if trapezius muscle activity amplitudes were higher during Near than during Far focusing periods, paired sample t-test were run on Far normalized values (%Far). T-tests were run for each viewing condition and group.
- To test whether eye lens accommodation response and trapezius muscle activity co-vary in time during the Near-Far task, the cross-correlation function (R(time shift)) was computed for each viewing condition and group [24].

This cross-correlation function computes the correlation coefficients between two signals by time shifting one of the signals. When the time shift is zero, the original signals are synchronized in time and the cross correlation function, R(time shift = 0), equals Pearson’s correlation coefficient, i.e. if two signals are identical, R(0) = 1. If these signals co-vary in time, R(time shift) will differ significantly from zero [25,26].

Cross-correlation functions were computed for each subject and each viewing condition. Mean cross-correlation curves within viewing condition and group were calculated. Group differences (between controls and patients) in maximal cross-correlation within viewing condition were analysed using independent sample t-test. One sample t-test was then applied to determine whether the maximal cross-correlation differed from zero within viewing condition and group.

The time shift associated with the maximal cross-correlation was estimated from the mean cross-correlation curves within viewing condition and group. To test whether this time shift differed significantly from zero, time shift was examined at the lower 95% confidence limit of the maximal cross-correlation from the mean cross-correlation curve, and on both sides of the maximal cross-correlation, as described by Li & Cadwell (1999) [26]. This was done within viewing condition and group.

To evaluate hypothesis (ii); for viewing conditions requiring more eye lens accommodation, the effect on trapezius muscle activity and the co-variation in time described in hypotheses (i) should be more pronounced, was analysed with the paired sample t-test. The test was run pairwise within viewing condition and group on both trapezius muscle activity (in %Far) during Near focusing, and on the maximal cross-correlation.

## Results

### Accommodation response and reaction time

Five subjects (two controls and three patients) were excluded from the accommodation mean values within the negative viewing condition and three subjects (one control and two patients) from ditto within the neutral and positive conditions. The exclusion was due to noisy data from the auto refractor and too few samples when calculating individual mean values. Fig 2 shows an example of how the accommodation response changes during the Near-Far task when the subject changes his/hers focus from the Near to the Far target.

**Fig 3:**
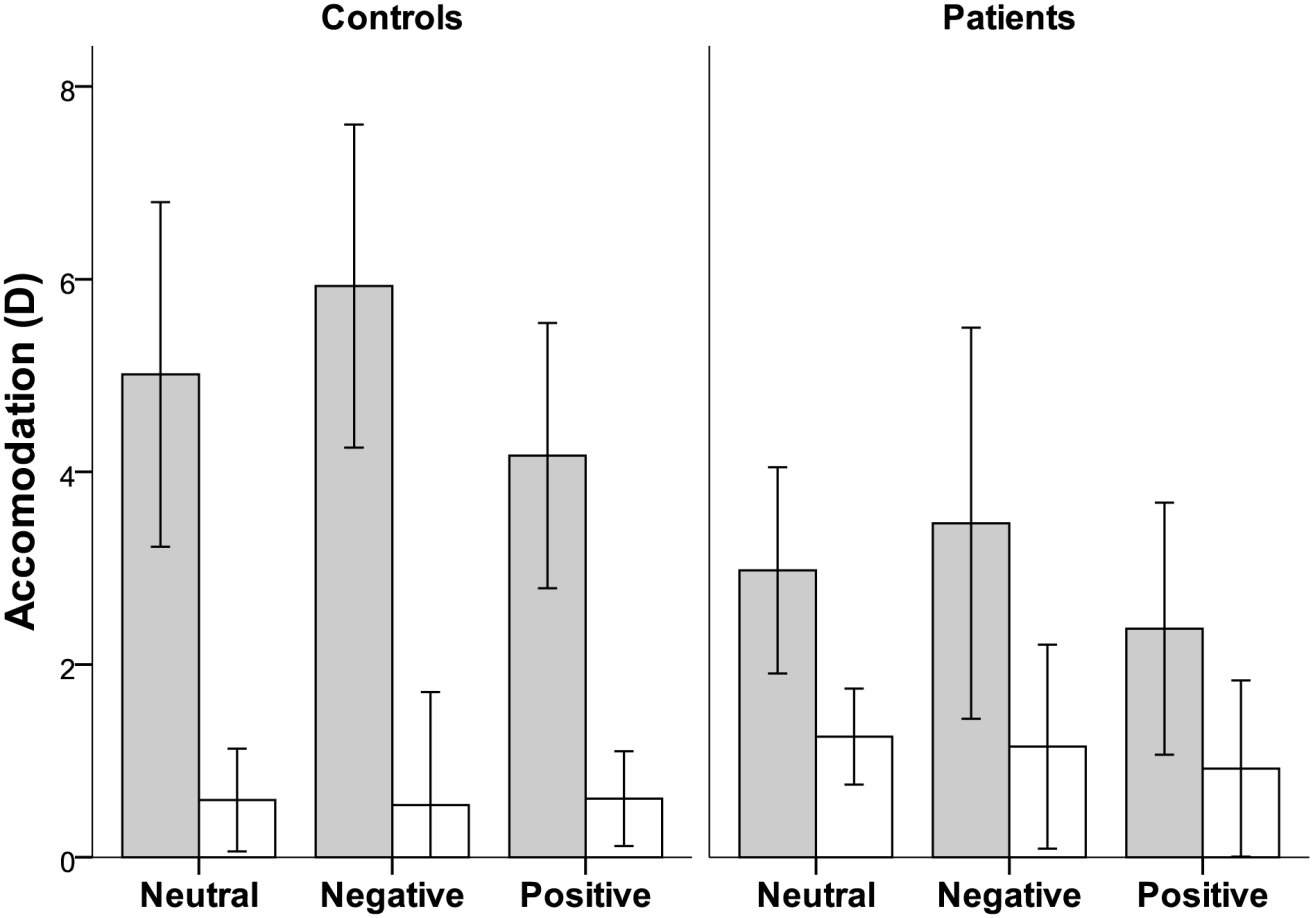
Accommodation response. Mean accommodation (and 95% confidence intervals), for the control group and for the patient group, from the periods with Near (grey bars) and Far (white bars) focusing for the three viewing conditions.

Mean accommodation from the periods with Near and Far focusing within viewing condition and group are shown in Fig 3. There was a significant difference between groups in accommodation response during Near focusing within the negative viewing condition, with higher accommodation responses in the control group (p = 0.049). No other group differences in accommodation were evident.

**Fig 4:**
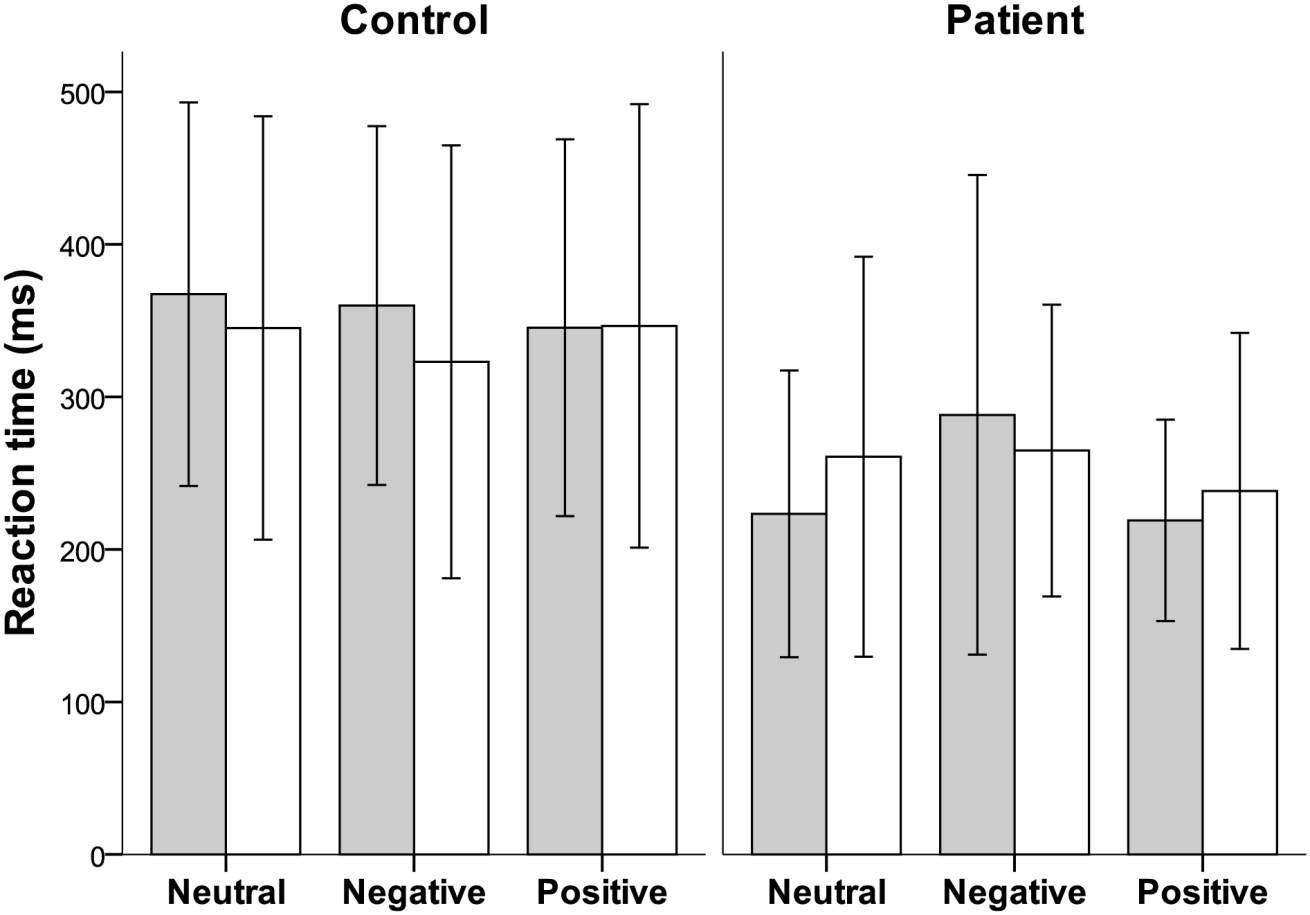
Reaction time. Mean reaction times (and 95% confidence intervals), for the control group and for the patient group for target shifts from Near-to-Far (grey bars) and from Far-to-Near (white bars) for the three viewing conditions.

The mean reaction times when changing focus from Near-to-Far and from Far-to-Near within viewing condition and group are displayed in Fig 4. No significant differences were found between groups for reaction times.

### Trapezius muscle activity

The mean trapezius muscle activity (in %RVE) (95% confidence intervals in brackets) during the Far focusing periods for the controls were: neutral condition 2.50 [0.84, 4.16], negative condition 2.71 [1.08, 4.35], positive condition 2.53 [0.98, 4.09], and for the patients: neutral condition 1.82 [0.79, 2.86], negative condition 2.36 [0.72, 3.99], positive condition 1.74 [0.28, 3.21].

**Fig 5:**
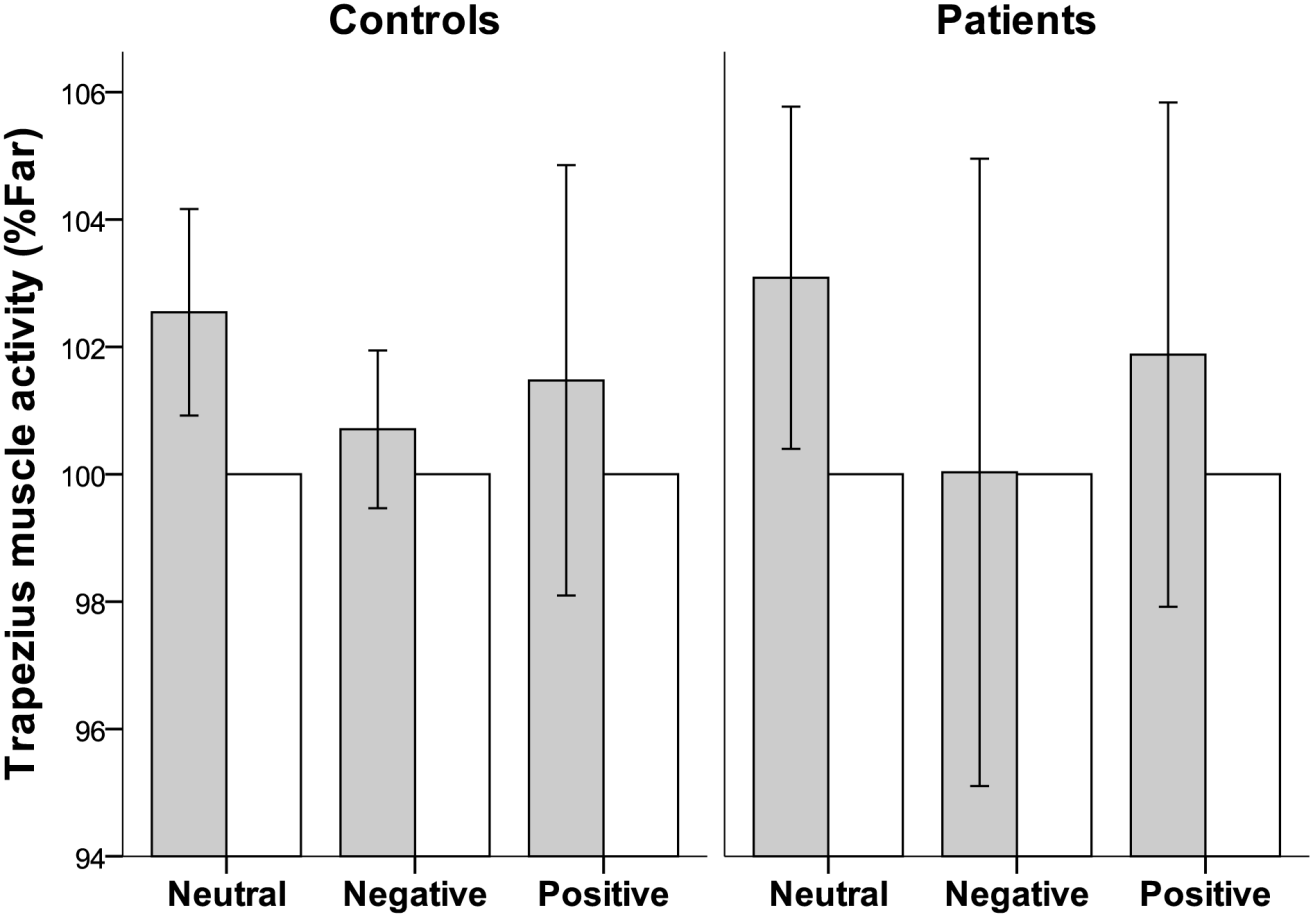
Trapezius muscle activity. Mean trapezius muscle activity (in %Far, with 95% confidence intervals) for the control group (n = 14) and for the patient group (n = 12) for the three viewing conditions. The grey bars shows muscle activity during near focusing and the white bars muscle activity during far focusing. The RMS EMG value from the Near periods for a specific viewing condition, was normalised to the mean RMS EMG value from the Far periods for the same viewing condition and expressed in %Far. In this way the value of the Far period was set to 100%Far for each trial lens. The white bars represents Far.

The trapezius muscle activity values (in %Far) within viewing condition and group are displayed in Fig 5. No significant differences were found between groups for trapezius muscle activity. Trapezius muscle activity was higher during Near than during Far focusing for the neutral viewing condition for both controls (p = 0.007) and patients (p = 0.034). For the negative and positive viewing conditions there were no significant differences in either group (p > 0.1).

### Cross-correlations

**Fig 6:**
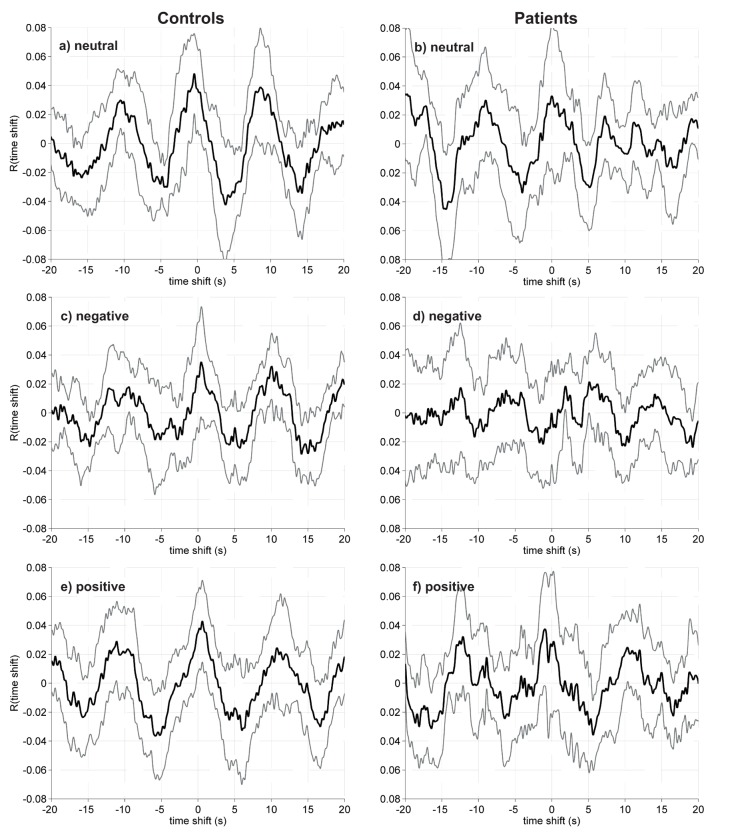
Cross-correlation functions. Mean cross-correlation curves and the 95% confidence intervals (grey lines). The curves to the left shows the control group (n = 14), and the curves to the right shows the patient group (n = 12). a—b) neutral viewing condition, c—d) negative viewing condition, e—f) positive viewing condition.

Mean percentage of valid samples for accommodation (or relative time used for the cross-correlation analyses) from the auto refractor for the Near-Far task within viewing condition were for the control group: neutral 77% negative 77%, and positive 79%, and for the patients: neutral 83%, negative 78%, and positive 84%. The mean maximal cross-correlation and time shift (in seconds) for the two groups are displayed in Table 2, and the cross-correlation curves for the two groups are shown in Fig 6. There was a significant difference in maximal cross-correlation between groups within the positive viewing condition with higher cross-correlation in the control group (p = 0.008). No other differences between groups were evident.

**Table 2:** Results from the cross-correlation analyses.

Group	Trial lens	Maximal cross correlation	Time shift in seconds [CI]
Control group	Neutral	0.048 (p = 0.002)	-0.44 [-2.2–0.78]
(n = 14)	Negative	0.035 (p = 0.066)	0.44 [-1.65–3.0]
	Positive	0.038 (p = 0.012)	0.60 [-1.08–1.14]
Patient group	Neutral	0.028 (p = 0.185)	0.00 [-1.8–3.6]
(n = 12)	Negative	-0.008 (p = 0.674)	*
	Positive	0.029 (p = 0.213)	-0.92 [-2.4–1.3]

Cross-correlation analyses were run within groups (controls and patients) and viewing conditions. One sample t-test tested if maximal cross-correlation (R(time shift)) differed from zero (p-value in brackets). Viewing conditions: neutral = no lenses, negative = -3.5 D lenses, positive = 3.5 D lenses. CI = 95% confidence intervals.

* Time shift not possible to estimate due to the shape of the curve (Fig 6d).

For the control group the maximal cross-correlation differed significantly from zero for the neutral and the positive viewing conditions, but not for the negative condition. In the patient group none of the maximal cross-correlations differed significantly from zero. None of the estimated time shifts differed significantly from zero; neither in the control group nor in the patient group (Table 2).

### Ciliary muscle activity and its effect on trapezius muscle activity and maximal cross-correlation

Results of the paired sample t-test within the control group showed that trapezius muscle activity during Near focusing periods (in %Far) was significantly higher for the neutral than for the negative viewing condition (p = 0.013), but there were no significant differences in any other case (p > 0.5, cf. Fig 5). In the patient group there were no significant differences in muscle activity among the viewing conditions (p > 0.3).

For the maximal cross-correlation (R(time shift)) there were no significant differences among viewing conditions, neither within the control group (p > 0.441), nor within the patient group (p > 0.22).

## Discussion

During the Near-Far task the amplitudes of the trapezius muscle activity were higher when focusing on a nearby than on a distant target. This difference was significant for the neutral viewing condition for both controls and patients. Furthermore, there was a significant cross-correlation between trapezius muscle activity and eye lens accommodation during the Near-Far task within the control group for the neutral and the positive viewing conditions, but not for the negative condition. In the patient group none of the maximal cross-correlations differed significantly from zero. There were no signs of pronounced effects for viewing conditions requiring more eye lens accommodation, neither on the trapezius muscle activity nor on the cross-correlation.

### The cross-correlation

The cross-correlation between trapezius muscle activity and eye lens accommodation during the Near-Far task within the control group for the neutral and positive viewing conditions was small, but statistically significant. None of the time shifts were significantly different from zero. The cross-correlation curves within the control group for the neutral and the positive viewing conditions exhibited a wave pattern with maxima occurring at times that were consistent with the mean interchange time between Near and Far focusing periods (5 s) (Fig 6a and 6e). This pattern is a strong indicator of a significant relation between the signals. For the negative viewing condition within the control group, the cross-correlation curve showed a similar pattern, even though the maximal cross-correlation did not differ significantly from zero (Fig 6c). For the patients, none of the maximal cross-correlations differed significantly from zero, although the neutral and positive curves (Fig 6b and 6f) exhibited a similar wave pattern as for the curves of the control group.

A theory mentioned in the introduction, suggests that a direct link between accommodation and trapezius muscle activity could involve the ciliary muscles: When clear vision is needed an efferent signal is sent to the ciliary muscles to alter the refractive power of the eye lens, a copy of this signal could be sent to the trapezius muscle and/or other muscles in the neck/shoulder area in order to stabilize gaze (cf. Richter et al., 2010). If this link exists, we would likely see a pronounced effect both on trapezius muscle activity and on cross-correlation for the negative viewing condition which required more activity in the ciliary muscles. In this sample of data there was no sign of a pronounced effect within the negative viewing condition. Instead it was the neutral viewing condition, with a slightly lower mean accommodation response than the negative condition (cf. Fig 4), that showed significant effects on both trapezius muscle activity and on cross-correlation. In addition, when comparing increases in trapezius muscle activity during Near focusing periods among viewing conditions, the neutral condition showed significantly higher increases compared to the negative condition within the control group.

### Accommodative and convergent demands

A study by Kapoula et al. (2006) showed decreased body sway during near focusing when the eyes converged more, than during far focusing with less demands on convergence. One suggestion arising from those results was that oculomotor signals and gaze related activity in the neck muscles are involved in postural stability [27]. A more recent study from the same group investigated the effect of spherical and prisms lenses’ on postural stability [28]. They found that prism lenses induced the highest degree of body sway (destabilisation), but interestingly, they also found increased body sway with spherical minus lenses, and the body sway was most pronounced with the -1 D lens, compared with the -3 D lens. Both the prism lenses and spherical minus lenses created a conflict (incongruence) between convergence and accommodation, and it appeared that the subjects were able to handle the conflict more effectively with the stronger conflict (-3 D lenses) than with a weaker one (-1 D lenses). The authors suggested that accommodation and accommodative vergence induced by lenses could modify activity in the neck muscles involved in postural control [28].

In line with the above suggestion, and since the visual system has little tolerance to errors in convergence [29], a relevant question would be if incongruence affects not only instability [28], but also may increase muscular activity. In our study, the negative trial lenses put conflicting demands on the visual system during the Near-Far task. When the negative trial lenses interrupts the line of sight, the eye lens needs to accommodate to reduce the blur, and at the same time keep the convergence (or the direction of gaze) constant, although the trial lenses gives an impression that the target is further away. Thus, the negative viewing condition is incongruent for the visual system. Hence, the negative viewing condition did not only give the highest average accommodation response, but it also induced incongruence. If incongruence would contribute to the muscular activity increase while going from Far to Near focusing, a higher increase would be expected for the negative viewing condition than for the other conditions. Again, that was not the case in the present results. The lack of effect might derive from the fact that incongruence was present both during near and during far focusing with the negative lenses, and that the presence of incongruence per se is the important factor, and not the level of incongruence (cf. [28]).

As described above, the negative viewing condition created incongruence between vergence and accommodation which altered the natural couplings between accommodation and convergence. The positive condition was also hard for the visual system, a positive +3.5 D blur is almost impossible to overcome. Fig 3 shows that there are small differences in the accommodative responses among the conditions, and that the responses for the negative and the positive condition differ from the accommodative stimulus. This suggests that the subjects primarily converged to targets, and that the accommodation responses derive mainly from convergence accommodation [30]. This suggestion is supported by previous findings where subjects focused for five minutes on a nearby target through -3.5 D lenses [11], where the individual convergence responses were close to stimuli, but the individual accommodative responses in most cases were lower than the stimuli and lower than expected (see Fig 4a in Richter et al. 2010 [11]). Taken together, the negative and positive trial lenses only induced small differences in the level of accommodation responses during the Near-Far task. In future experiments, natural congruent viewing conditions are recommended, and in this experiment it would have been better to use trials with different distances to the nearby target, where a closer distance should induce a higher level of accommodation, than to use lenses in order to vary the accommodative responses.

### Eye-neck (head) stabilization during the Near-Far task

Even though the attempt to create increased and decreased levels of accommodation failed to show any linear association between accommodation response and trapezius muscle activity, a direct link between eye lens accommodation and trapezius muscle activity cannot be ruled out. Another explanation for the increase in muscular activity during near focusing could be the necessity for eye-neck (head) stabilization; a head movement in the frontal plane results in a relatively large change in visual degrees when focusing on a nearby target, while for a distant target, the visual angle will remain almost unchanged. This suggestion is analogous to the coordination of eye and neck muscles when shifting gaze [31]. Individuals with whiplash-associated disorders suffer from visual deficits and demonstrate changes in eye-neck coordination, e.g., altered activity of the deep muscles that stabilize the neck [32]. According to this suggestion, the non-significant cross-correlation results may also be a sign of an altered function in the visual stabilisation process involving eye-neck (head) interactions in subjects with a history of asthenopic symptoms and concurrent self-reported neck problems.

### Methodological considerations

During the experiment, the subjects were seated in an office chair in a slightly leaned back position supported by a combined head and backrest adjusted to their morphology. They were instructed to keep this posture throughout the experiment. This was necessary in order to measure eye lens accommodation with the auto refractor. Consequently, the head and neck were externally stabilized, and therefore there was no real need for increased muscle activity in muscles involved in eye-neck (head) stabilization. Still, significant muscular activity increases were seen. These increases might have been larger without the external support.

The upper trapezius muscle can be monitored easily with surface EMG, is a common site for work-related pain, and has been the focus of much research on EMG signals in relationship to sedentary work, such as computer work [33]. However, the trapezius muscle is not primarily involved in stabilization of the neck, but is considered mainly to stabilize and move scapula [33,34]. If the cross-correlation between trapezius muscle activity and eye lens accommodation observed here is due to need for stabilization of the head and neck during the demanding Near-Far task, there should be a similar or even more pronounced cross-correlation between accommodation and muscles acting as primary stabilizers of the neck (i.e. deep cervical flexor and extensor muscles [35,36]). Unfortunately, the deep cervical muscles are difficult to monitor non-intrusively [37].

The controls had significantly higher accommodation responses during Near focusing within the negative condition than the patients. Here it should be noted that the patients near point of accommodation was lower, which resulted in a slightly longer mean distance to the near target (Table 1). The origin of the visual symptoms in the patient group was mixed, and the difference in accommodation response between groups was most likely due to the origin of the asthenopic symptoms in the patient group, where several of them had accommodative insufficiency.

The patients also all had self-reported unspecific neck/shoulder discomfort of unknown origin. This heterogeneity in the patient group might have affected the results of the study. In the patient group, the maximal cross-correlation was not significantly above zero for any condition, but the group mean cross-correlation curves showed the characteristic waveform in the neutral and positive conditions. In addition to the suggestion above, that the non-significant cross-correlation results in the patient group might be a sign of an altered function in the visual stabilisation process involving eye-neck (head) interactions, it may also be due to the heterogeneity in the group, and/or a lack of power in the analysis. In retrospect, it would have been an advantage to (a) increase the number of subjects in the patient group to increase power in the analysis, (b) to have a homogenous patient group with similar visual problems, and (c) to use a standardised physical examination method [38,39]) to diagnose the disorder of the neck and upper limb in order to also have homogeneity in the neck/shoulder symptoms within the patient group.

Accommodation to a visual target involves an average reaction time of approximately 0.5 s (latency 0.3–0.4 s, duration 0.2–1.0 s) [40]. Mean reaction times documented here are between 0.2–0.4 s (Fig 4) and are within the expected magnitude. In the neutral viewing condition, the mean distance to the near target for all subjects was 28 cm and the distance to the far target was 3 m, corresponding to 3.6 D and 0.3 D, respectively. As can be read from Fig 3, mean accommodation response for the neutral trial lens is close to the expected values both during near and far focusing. The documented reaction times and the accommodation responses within the neutral viewing condition validates the procedure used to measure accommodation [22].

Under working conditions that induce low demands on muscles, muscle activity amplitude varies slowly with time. To isolate the effect of Near and Far focusing on trapezius muscle activity, we used short time periods in our task, which effectively reduces the variance in comparison to longer Near and Far periods. Moreover, muscle activity during Near was normalised to the adjacent Far period, to obtain individual Near-Far ratios prior to the averaging.

All average maximal R-values of the cross-correlation curves were less than 0.05 (cf. Table 2). Both the trapezius muscle activity and accommodation signals include random components, mostly due to biological variance for muscle activity, and technical noise for accommodation. These random components are not expected to be correlated, and are of higher frequency than the changes in focus (which had a mean frequency of 0.2 Hz). In this investigation a 3-Hz low-pass filter was applied to both signals. A use of a low-pass filter with a lower cut-off frequency would probably have increased the peak cross-correlation values.

## Conclusion

Our present findings reveal that trapezius muscle activity is higher when focusing on a nearby than on a distant target, and that there is a co-variation in time between eye lens accommodation and trapezius muscle activity during dynamic changes in focus between a nearby and a far target. The effect on trapezius muscle activity was significant for the neutral viewing condition for both the control group and the patient group. In addition, the cross-correlation was significant for the neutral and the positive viewing conditions within the control group. A direct link, from the accommodation/vergence system to the trapezius muscles cannot be ruled out, but the connection may also be explained by an increased need for eye-neck (head) stabilization when focusing on a nearby target as compared to a more distant target.

## Supporting Information

S1 File Dataset. (XLSX)
